# Production of propionate using metabolically engineered strains of *Clostridium saccharoperbutylacetonicum*

**DOI:** 10.1007/s00253-022-12210-8

**Published:** 2022-10-25

**Authors:** Tina Baur, Alexander Wentzel, Peter Dürre

**Affiliations:** 1 Institut für Mikrobiologie und Biotechnologie, Universität Ulm, Albert-Einstein-Allee 11, 89081 Ulm, Germany; 2 Department of Biotechnology and Nanomedicine, SINTEF Industry, Richard Birkelands vei 3, 7034 Trondheim, Norway

**Keywords:** *Clostridium saccharoperbutylacetonicum*, Propionate production, Acrylate pathway, Fluorescence-activating and absorption-shifting tag, Promoter activities, Two-plasmid system

## Abstract

**Abstract:**

The carboxylic acid propionate is a valuable platform chemical with applications in various fields. The biological production of this acid has become of great interest as it can be considered a sustainable alternative to petrochemical synthesis. In this work, *Clostridium saccharoperbutylacetonicum* was metabolically engineered to produce propionate via the acrylate pathway. In total, the established synthetic pathway comprised eight genes encoding the enzymes catalyzing the conversion of pyruvate to propionate. These included the propionate CoA-transferase, the lactoyl-CoA dehydratase, and the acryloyl-CoA reductase from *Anaerotignum neopropionicum* as well as a *D*-lactate dehydrogenase from *Leuconostoc mesenteroides* subsp. *mesenteroides*. Due to difficulties in assembling all genes on one plasmid under the control of standard promoters, the P_*tcdB*_-*tcdR* promoter system from *Clostridium difficile* was integrated into a two-plasmid system carrying the acrylate pathway genes. Several promoters were analyzed for their activity in *C. saccharoperbutylacetonicum* using the fluorescence-activating and absorption-shifting tag (FAST) as a fluorescent reporter to identify suitable candidates to drive *tcdR* expression. After selecting the lactose-inducible P_*bgaL*_ promoter, engineered *C. saccharoperbutylacetonicum* strains produced 0.7 mM propionate upon induction of gene expression. The low productivity was suspected to be a consequence of a metabolic imbalance leading to acryloyl-CoA accumulation in the cells. To even out the proposed imbalance, the propionate-synthesis operons were rearranged, thereby increasing the propionate concentration by almost four-fold. This study is the first one to report recombinant propionate production using a clostridial host strain that has opened a new path towards bio-based propionate to be improved further in subsequent work.

**Key points:**

*• Determination of promoter activities in C. saccharoperbutylacetonicum using FAST.*

*• Implementation of propionate production in C. saccharoperbutylacetonicum.*

*• Elevation of propionate production by 375% to a concentration of 3 mM.*

**Supplementary Information:**

The online version contains supplementary material available at 10.1007/s00253-022-12210-8.

## Introduction

**Fig. 1:**
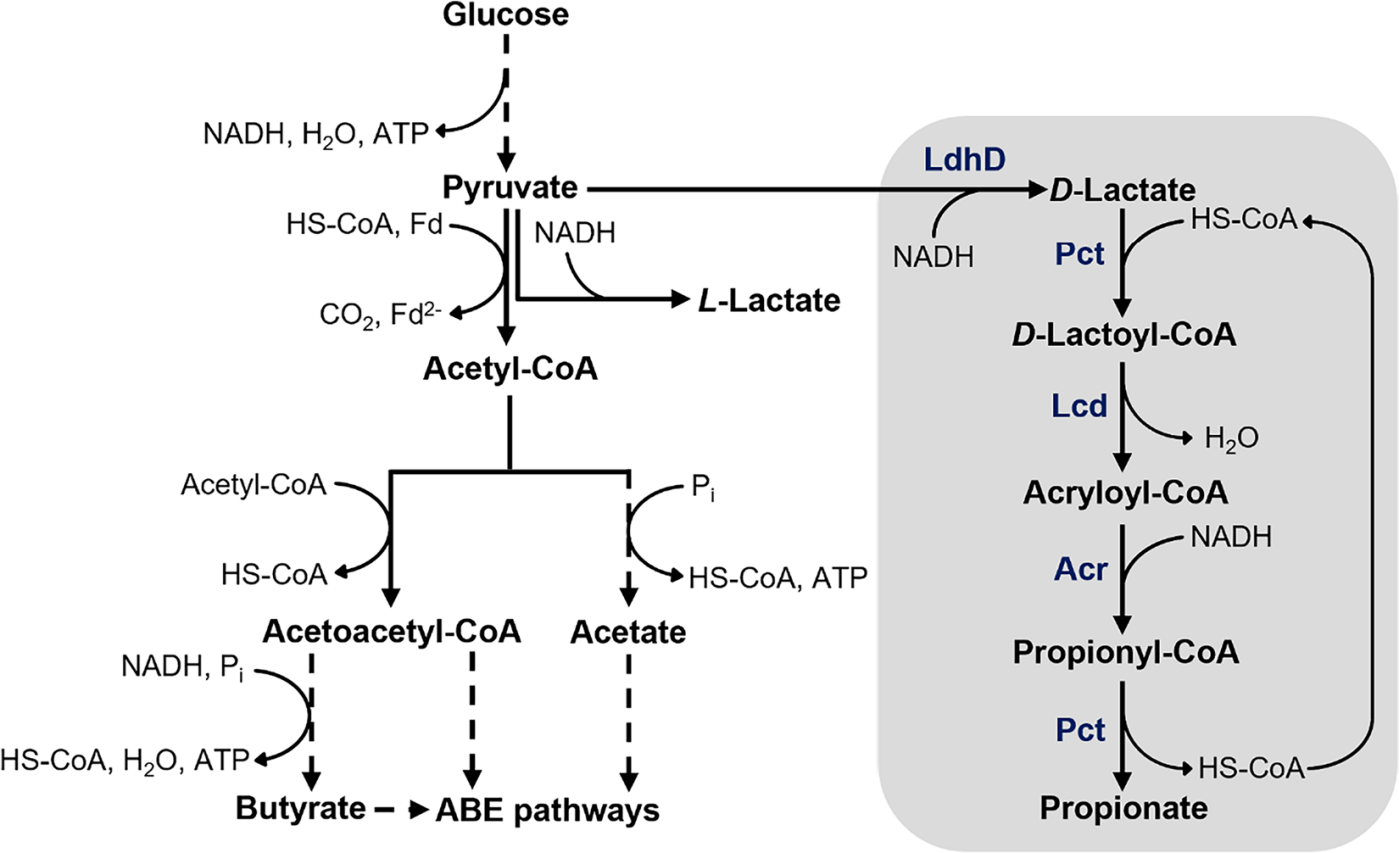
Schematic overview of glycolytic and acidogenic pathways in *C. saccharoperbutylacetonicum* based on Jones and Woods (1986) coupled with acrylate pathway for propionate production (grey box; based on Hetzel et al. 2003) from *An. neopropionicum* and *D*-lactate dehydrogenase from *L. mesenteroides* subsp. *mesenteroides* (stoichiometrically incorrect). LdhD, *D*-lactate dehydrogenase (LEUM_1756); Pct, propionate CoA-transferase (CLNEO_17700); Lcd, lactoyl-CoA dehydratase (CLNEO_17730-17710); Acr, acryloyl-CoA reductase (CLNEO_21740-21760); ABE, acetone-butanol-ethanol

Propionate is a valuable platform chemical with a wide range of applications. Due to its antimicrobial activity, it is mostly used as a food and feed preservative, an ingredient in cleaning agents, or as an herbicide. It also gains increasing importance in the production of pharmaceuticals, plastics, and cosmetics (Gonzalez-Garcia et al. 2017; Samel et al. 2018). Moreover, it is considered an important precursor chemical as it is often esterified with short-chain alcohols, olefins, or acetylenes to yield corresponding alcohol or vinyl esters, which themselves have versatile applications (Samel et al. 2018). Propionate synthesis is currently achieved via chemical processes, i.e., the carbonylation of ethylene or the oxidation of propionaldehyde (Samel et al. 2018). However, bio-based approaches using bacteria as cell factories for propionate production from cheap or waste-derived substrates become increasingly attractive as sustainable alternatives to petrochemical production (Stowers et al. 2014). Although not commercially profitable yet, the desire to achieve an environmentally friendly propionate production has led to multiple studies exploring the capabilities of bacteria in that regard. There are different bacterial species that can naturally produce propionate from a range of substrates and via different pathways, e. g. *Propionibacterium* sp. via the Wood-Werkman cycle, or *Clostridium* and *Megasphaera* sp. via the acrylate pathway (Gonzalez-Garcia et al. 2017). Especially *Propionibacterium* sp. such as *Propionibacterium acidipropionici* and *Propionibacterium freudenreichii* have been studied extensively to develop fermentation strategies that allow the turnover of cheap substrates such as glycerol or molasses to propionate (Dishisha et al. 2012; Feng et al. 2011). Furthermore, these bacteria have also been engineered to improve propionate yields and overcome typical fermentation obstacles such as low acid tolerance (Jiang et al. 2015; Wang et al. 2015). Aside from propionibacteria, other non-native propionate-producing microorganisms have been engineered for propionate production, including *Escherichia coli* (Akawi et al. 2015; Gonzalez-Garcia et al. 2020; Kandasamy et al. 2013), *Lactobacillus plantarum* (Balasubramanian and Subramanian 2019), and *Pseudomonas putida* (Ma et al. 2020; Mu et al. 2021). Surprisingly, clostridia have never been considered hosts for recombinant propionate production although they are organisms with a versatile metabolism enabling them to use diverse carbon sources, including lignocellulosic hydrolysates and waste-derived substrates, and convert them into various products (Cho et al. 2015; Tracy et al. 2012). Furthermore, multiple tools are available to genetically modify clostridia for optimized production of native or recombinant compounds thus making them promising host strains for the production of commodity chemicals such as ethanol, isopropanol, 2,3-butanediol, or fatty acid esters (Cho et al. 2015; Feng et al. 2021). *Clostridium saccharoperbutylacetonicum* is a well-characterized solventogenic bacterium, which is genetically accessible and has high growth rates in favorable medium. Since it is a known hyper-butanol producer, it has mostly been employed for butanol production (Jiménez-Bonilla et al. 2021). However, *C. saccharoperbutylacetonicum* has also successfully been used for the production of hydrogen (Singh et al. 2019), isopropanol (Wang et al. 2019), 1,3-butanediol (Grosse-Honebrink et al. 2021), as well as caproate and hexanol (Wirth and Dürre 2021), thus highlighting its potential as a host for production of recombinant compounds. Here, we report the approach to convert *C. saccharoperbutylacetonicum* into a propionate producer by the implementation of the acrylate pathway from *An. neopropionicum* and a *D*-lactate dehydrogenase from *L. mesenteroides* subsp. *mesenteroides* (Fig. 1). For that purpose, a two-plasmid system harboring two propionate-synthesis operons (PSOs) was constructed, and gene expression was controlled by the sigma factor-inducible P_*tcdB*_ promoter from *C. difficile*. In order to identify promoters that are suitable for mediation of gene expression in *C. saccharoperbutylacetonicum*, a promoter study using FAST was conducted.

## Materials and methods

### Bacterial strains and cultivation

Bacterial strains and plasmids used are listed in Table 1. *E. coli* XL1-Blue MRF´ was used for cloning purposes and cultivated aerobically under constant shaking (180 rpm) at 37 °C in Luria–Bertani (LB) medium (Green and Sambrook 2012) supplemented with respective antibiotics. For the preparation of chemically competent cells, *E. coli* was grown in Super Optimal Broth (SOB; Hanahan 1983) and cultivated at 18 °C with shaking (100 rpm). *C. saccharoperbutylacetonicum* N1-4(HMT) DSM 14923 was used as a production host for propionate and cultivated under strictly anaerobic conditions at 30 °C in complex (clostridial growth medium, CGM) or minimal medium (optimized synthetic medium, OMS). Both media were prepared as previously described by Wirth and Dürre (2021); however, sodium sulfide was omitted from OMS. When *C. saccharoperbutylacetonicum* was cultivated on solid medium, CGM was supplemented with 1.5% agar (w v^−1^) and antibiotics. The medium was prepared and poured within an anaerobic cabinet with a N_2_:H_2_ (95:5%) gas atmosphere using anaerobic water. Cells of *C. saccharoperbutylacetonicum* grown on solid CGM were cultivated in an incubator located in the anaerobic cabinet at 32 °C. Antibiotics used to select recombinant bacterial strains were supplemented to the following concentrations (per milliliter): 30 µg chloramphenicol, 10 µg clarithromycin, 300 µg erythromycin, 10 µg tetracycline, 40 µg thiamphenicol.

**Table 1:** Bacterial strains and plasmids used in this study

Bacterial strain or plasmid	Relevant characteristics	Origin
*C. saccharoperbutylacetonicum* N1-4(HMT) DSM 14923	Type strain	DSMZ^a^ GmbH, Brunswick, Germany
*E. coli* XL1-Blue MRF´	Δ(*mcrA*)183 Δ(*mcrCB*-*hsdSMR*-*mrr*)173*endA1 supE44 thi*-1 *recA1 gyrA96 relA1 lac*(*F’proAB lacI*^*q*^ZΔM15 Tn10 (Tet^R^))	Agilent Technologies, Santa Clara, CA, USA
pMTL82251	Em^r^ (*ermB*), ColE1 ori^–^, pBP1 ori^+^, *lacZα, traJ*	Heap et al. 2009
pMTL82251_LL	pMTL82251, *ldhD* from *L. mesenteroides* subsp. *mesenteroides*, *lcdC*, *lcdA*, and *lcdB* from *An. neopropionicum*	This study
pMTL82251_P_*tet*__LL	pMTL82251_LL, P_*tet*_	This study
pMTL82251_P_*tet*__LL_tcdR	pMTL82251_P_*tet*__LL, *tcdR* from *C. difficile*	This study
pMTL82251_P_*bgaL*__LL_tcdR	pMTL82251_P_*tet*__LL_tcdR with P_*bgaL*_ from *Clostridium* *perfringens* instead of P_*tet*_	This study
pMTL82251_P_*bgaL*__LPTT	pMTL82251, P_*bgaL*_ from *C. perfringens*, *ldhD* from *L. mesenteroides* subsp. *mesenteroides*, *pct* from *An. neopropionicum*, and 2 × *tcdR* from *C. difficile*	This study
pMTL83151	Cm^r^ (*catP*), ColE1 ori^–^, pCB102 ori^+^, *lacZα, traJ*	Heap et al. 2009
pMTL83151_PA	pMTL83151, *pct*, *acrC, acrB,* and *acrA* from *An. neopropionicum*	This study
pMTL83151_gusA_P_*bgaL*_	pMTL83151, P_*bgaL*_ from *C. perfringens, gusA* from *E. coli*	Beck et al. 2020
pMTL83151_P_*tet*__LLP	pMTL83151, P_*tet*_, *ldhD* from *L.* *mesenteroides* subsp. *mesenteroides*, *lcdC*, *lcdA*, *lcdB*, and *pct* from *An. neopropionicum*	This study
pMTL83151_P_*tet*__LLPA	pMTL83151_P_*tet*__LLP, *acrC*, *acrB*, *and acrA* from *An. neopropionicum*	This study
pMTL83151_P_*tcdB*__PA	pMTL83151_PA, P_*tcdB*_ from *C. difficile*	This study
pMTL83151_P_*tcdB*__L_P_*tcdB*_	pMTL83151, P_*tcdB*_ from *C. difficile*, *lcdC*, *lcdA*, and *lcdB* from *An. neopropionicum*	This study
pMTL83151_P_*tcdB*__L_P_*tcdB*__AA	pMTL83151_P_*tcdB*__L_P_*tcdB*_, 2 × *acrC*, *acrB*, and *acrA* from *An. neopropionicum*	This study
pMTL83151_P_*tet*__3-HP_opt	pMTL83151, P_*tet*_, *ldhD* from *L. mesenteroides* subsp. *mesenteroides*, *lcdC*, *lcdA*, *lcdB*, and *pct* from *An. neopropionicum*, *ehy_opt* (*ehy* from *Chloroflexus aurantiacus* codon-optimized for *Acetobacterium woodii*; sequence provided in Fig. S5)	Beck 2020
pMTL83251	Em^r^ (*ermB*), ColE1 ori^–^, pCB102 ori^+^, *lacZα, traJ*	Heap et al. 2009
pMTL83251_P_*bld*__FAST	pMTL83251, P_*bld*_ from *C.* *saccharoperbutylacetonicum*, *feg* (FAST-encoding gene from *Halorhodospira halophila* codon-optimized for *Clostridium* *acetobutylicum*; Streett et al. 2019)	This study
pMTL83251_P_*bgaL*__FAST	pMTL83251, P_*bgaL*_ from *C. perfringens*, *feg*	Flaiz et al. 2021
pMTL83251_P_*lctB*__FAST	pMTL83251, P_*lctB*_ from *A. woodii*, *feg*	This study
pMTL83251_P_*pta-ack*__FAST	pMTL83251, P_*pta-ack*_ from *Clostridium ljungdahlii, feg*	Flaiz unpublished
pMTL83251_P_*thlA*__FAST	pMTL83251, P_*thlA*_ from *C. acetobutylicum*, *feg*	Flaiz et al. 2021
pMTL83251_P_*tcdB*__FAST	pMTL83251, P_*tcdB*_ from *C. difficile*, *feg*	This study
pMTL83251_P_*tcdB*__FAST_P_*bld*__tcdR	pMTL83251_P_*tcdB*__FAST, P_*bld*_ from *C. saccharoperbutylacetonicum*, *tcdR* from *C. difficile*	This study
pMTL83251_P_*tcdB*__FAST_P_*bgaL*__tcdR	pMTL83251_P_*tcdB*__FAST, P_*bgaL*_ from *C. perfringens*, *tcdR* from *C. difficile*	This study

^a^ German Collection of Microorganisms and Cell Cultures (DSMZ)

Growth experiments for propionate production as well as fluorescence determination were performed in 50 ml OMS in 125-ml Müller-Krempel bottles (Müller & Krempel AG, Bülach, Switzerland) supplemented with respective antibiotics. In the case of propionate production, 2-ml samples were withdrawn throughout the course of growth for the determination of substrate consumption and product formation. For determination of promoter activities, 0.5–2-ml samples were withdrawn at different stages of growth and processed as described in the “FAST reporter assays” section.

### Plasmid construction

Standard molecular cloning techniques were performed according to established protocols (Green and Sambrook 2012). Linearization of plasmids was performed using “FastDigest™ enzymes” (Thermo Fisher Scientific Inc., Waltham, MA, USA). Primers used for amplification of genes and promoters are listed in Table 2 and were synthesized by biomers.net GmbH (Ulm, Germany). Amplification of DNA fragments was performed using the “CloneAmp™ HiFi polymerase” (Takara Bio USA, Inc., Mountain View, CA, USA) or the “Phusion™ Green High-Fidelity DNA polymerase” (Thermo Fisher Scientific Inc., Waltham, MA, USA). Generated DNA fragments were purified using the “Zymoclean™ Gel DNA Recovery Kit” (ZYMO Research Corp., Irvine, CA, USA) according to the manufacturer´s instructions. All plasmids were constructed using either the “In-Fusion® HD Cloning kit” (Takara Bio USA, Inc., Mountain View, CA, USA) or the “NEBuilder® HiFi DNA Assembly Master Mix” (New England Biolabs® Inc., Ipswich, MA, USA) following manufacturer´s instructions. A total of 8–10 µl of cloning mixtures were finally used to transform chemically competent *E. coli* cells. Plasmid DNA from *E. coli* strains was isolated using the “Zyppy™ Plasmid Miniprep Kit” (ZYMO Research Corp., Irvine, CA, USA) following the manufacturer´s instructions.

**Table 2:** Primers used in this study. Restriction sites are underlined and bold

Primer	Sequence (5´ → 3´)	Restriction site
Inf_ldhD-lcdCAB_fw	GTCACGCGTCCATGGAGATC**CTCGAG**ATGAAGATTTTTGCTTACGG	***Xho*I**
Inf_ldhD-lcdCAB_rev	GCAGGCTTCTTATTTTTATG**GCTAGC**TTACAGCATTTCTACGAAG	***Nhe*I**
Inf_lcd_fw	TTATAGTAAAGGAGAAAAT**TCTAGA**ATGTACACAATGGGCGTTG	***Xba*I**
Inf_lcd_rev	AATTCATTAA**CCCGGG**ATAAAAATAAGAAGCCTGCAAATG	***Sma*I**
Inf_Ptet_fw	GACCGATCGGGCCCCCTGCA**GTCGAC**ATAAAAATAAGAAGCCTGCATTTG	***Sal*I**
Inf_Ptet_LL_rev	CGTAAGCAAAAATCTTCATC**CTCGAG**GTTCTCCTTTACTGCAGG	***Xho*I**
Inf_tetR-pct_fw	GTCACGCGTCCATGGAGAT**CTCGAG**TCAAGCCTTCATTTCCTTC	***Xho*I**
Inf_tetR-pct_rev	GTTCAAAAAAATAATGGC**GGCGCGCC**ATAAAAATAAGAAGCCTGCATTTG	***Sgs*I**
Inf_pct_fw	TTGAATATTAAAGGAGGGTT**CCATGG**ATGAGAAAGGTTCCCATAATTAC	***Nco*I**
Inf_pct_rev	ATCCTCTCTTTCAAGCCTTCATTTCCTTC	
Inf_pct-acrCBA_fw	GTCACGCGTCCATGGAGATC**CACGTG**ATGAGAAAGGTTCCCATAATTAC	***Eco*72I**
Inf_pct-acrCBA_rev	GCAGGCTTCTTATTTTTATG**GCTAGC**CTAGGCATTTTTTGTCTCTTTG	***Nhe*I**
Inf_acr_fw2	GAAGGAAATGAAGGCTTGA**CTCGAG**AGGAAGGATGAATGGAATGGACTTTTCATTAACGAG	***Xho*I**
Inf_acr_rev2	CAGGAAACAGCTATGACCGC**GCTAGC**CTAGGCATTTTTTGTCTCTTTGATTG	***Nhe*I**
Inf_acr_fw3	TTATAGTAAAGGAGAAAAT**CTCGAG**ATGGACTTTTCATTAACGAG	***Xho*I**
Inf_acr_rev3	CATCCTTCCT**AGATCT**CTAGGCATTTTTTGTCTCTTTG	***Bgl*II**
Inf_acr_fw4	CTAG**AGATCT**AGGAAGGATGAATGGAATG	***Bgl*II**
Inf_acr_rev4	GTTCAAAAAAATAATGGC**GGCGCGCC**ATAAAAATAAGAAGCCTGCAAATG	***Sgs*I**
Inf_tcdR_wRBS_fw	CTTCGTAGAAATGCTGTAAGAAGAGAGGAT**GCTAGC**ATGCAAAAGTCTTTTTATGAATTA	***Nhe*I**
Inf_tcdR_wRBS_rev	CAGGCTTCTTATTTTTATGG**GGATCC**TTACAAGTTAAAATAATTTTCATAGTCT	***Bam*HI**
Inf_tcdR_fw2	GAAGGCTTGAAAGAGAGGAT**GCTAGC**ATG	***Nhe*I**
Inf_tcdR_rev2	TCATAAAAAGACTTTTGCAT**GGATCC**ATCCTCTCTTTTACAAGTTAAAATAATTTTCATAGTC	***Bam*HI**
Inf_tcdR-fdx_fw	TACT**CCCGGG**ATGCAAAAGTCTTTTTATGAATTAATTG	***Sma*I**
Inf_tcdR-fdx_rev	ATTTCTTTAAATTCATTAA**CATATG**ATAAAAATAAGAAGCCTGCAAATG	***Nde*I**
Inf_Pbld_fw2	AGGAAACAGCTATGACCGC**GTCGAC**GATATTTCCCCCATAAGTAAAG	***Sal*I**
Inf_Pbld_rev2	TAAAAAGACTTTTGCAT**CCCGGG**TCCTCCTTATGATTTAAAAATTAATAAC	***Sma*I**
Inf_Pbld_FAST_fw	GACCGCGGCCGCTGTATC**CATATG**GATATTTCCCCCATAAGTAAAG	***Nde*I**
Inf_Pbld_FAST_rev	CAAATGCTACGTGTTCCAT**GGATCC**TCCTCCTTATGATTTAAAAATTAATAA	***Bam*HI**
Inf_PbgaL_fw	ACCGATCGGGCCCCCTGCAG**GTCGAC**GAGATGAAAAGTATTAGGGC	***Sal*I**
Inf_PbgaL_rev	CGTAAGCAAAAATCTTCATCC**CTCGAG**TACCCTCCCAATACATTTAAA	***Xho*I**
Inf_PbgaL_tcdR-fdx_fw	CAGGAAACAGCTATGACCGC**GTCGAC**GAGATGAAAAGTATTAGGGC	***Sal*I**
Inf_PbgaL_tcdR-fdx_rev	ACTTTTGCAT**CCCGGG**AGTACCCTCCCAATACATTTAAAAT	***Sma*I**
Inf_PlctB_FAST_fw	GACCGCGGCCGCTGTATCCA**CATATG**TCAGGACTTATCAAGTTTAAGTC	***Nde*I**
Inf_PlctB_FAST_rev	CCAAATGCTACGTGTTCCAT**GGATCC**ACTCGCCCTCCATTAAATTAATTA	***Bam*HI**
Inf_PtcdB_fw	GACCGATCGGGCCCCCTGCA**GTCGAC**TTAATGAATTTAAAGAAATATTTACAATA	***Sal*I**
Inf_PtcdB_rev	ATTATGGGAACCTTTCTCAT**TCTAGA**ATTTTCTCCTTTACTATAATATTTTTATTG	***Xba*I**
Inf_PtcdB_fw2	TTAT**CCCGGG**TTAATGAATTTAAAGAAATATTTACAATAGAAATC	***Sma*I**
Inf_PtcdB_rev2	GCAGGCTTCTTATTTTTATC**CTCGAG**ATTTTCTCCTTTACTATAATATTTTTATTG	***Xho*I**
Inf_PtcdB_FAST_fw	GACCGCGGCCGCTGTATCCA**CATATG**TTAATGAATTTAAAGAAATATTTACAATA	***Nde*I**
Inf_PtcdB_FAST_rev	CCAAATGCTACGTGTTCCAT**GGATCC**ATTTTCTCCTTTACTATAATATTTTTATTG	***Bam*HI**

To establish propionate production in *C. saccharoperbutylacetonicum*, respective genes encoding the key enzymes of the acrylate pathway from *An. neopropionicum* were used (Beck et al. 2016). These include the propionate CoA-transferase (*pct*; CLNEO_17700), the subunits of the lactoyl-CoA dehydratase (*lcdC, lcdA, lcdB*; CLNEO_17730-17710), and the subunits of the acryloyl-CoA reductase (*acrC*, *acrB*, *acrA*; CLNEO_21740-21760). Furthermore, the well-characterized *D*-lactate dehydrogenase gene from *L.* *mesenteroides* subsp. *mesenteroides* (*ldhD*; LEUM_1756; Li et al. 2012) was inserted into the PSO. Previously, all mentioned genes except for *acrA*, *acrB*, and *acrC* were assembled on plasmid pMTL83151_P_*tet*__3-HP_opt (Beck 2020) and controlled by the tetracycline-inducible promoter P_*tet*_ (comprises P_*xyl*_ from *Bacillus subtilis* as well as *tetR* and P_*tetR*_ from Tn*10* from *E. coli*; Zhang et al. 2000; Fagan and Fairweather 2011; Beck et al. 2020). The aforementioned genes including P_*tet*_ were PCR-amplified from pMTL83151_P_*tet*__3-HP_opt using primers Inf_tetR-pct_fw and Inf_tetR-pct_rev and subcloned in pMTL83151, which was linearized with *Xho*I and *Sgs*I (resulting in pMTL83151_P_*tet*__LLP). In a second step, genes *acrA*, *acrB*, and *acrC* were amplified from genomic DNA of *An.* *neopropionicum* DSM 3847 using primers Inf_acr_fw2 and Inf_acr_rev2 and inserted into pMTL83151_P_*tet*__LLP digested with *Xho*I and *Not*I to yield pMTL83151_P_*tet*__LLPA. This plasmid could successfully be constructed; however, DNA concentration was low, and when the respective *E. coli* strain was inoculated to harvest enough DNA for transformation of *C. saccharoperbutylacetonicum*, the plasmid was altered. Therefore, genes encoding the acrylate pathway were divided into two PSOs and assembled on two plasmids, which were based on vectors pMTL83151 and pMTL82251 (Heap et al. 2009). PSO1 consisted of genes *pct*, *acrC*, *acrB*, and *acrA* (pMTL83151_PA), PSO2 harbored *ldhD*, *lcdC*, *lcdA*, and *lcdB* (pMTL82251_LL). For cloning of pMTL83151_PA and pMTL82251_LL, plasmids pMTL83151 and pMTL82251 were digested using enzymes *Xho*I and *Nhe*I and ligated with PCR-amplified fragments *pct*-*acrCBA* (from pMTL83151_P_*tet*__LLPA; primers Inf_pct-acrCBA_fw and Inf_pct-acrCBA_rev) and *ldhD*-*lcdCAB* (from pMTL83151_P_*tet*__LLP; primers Inf_ldhD-lcdCAB_fw and Inf_ldhD-lcdCAB_rev), respectively. To control the expression of both PSOs, the P_*tcdB*_-*tcdR* promoter system from *C. difficile* was selected since this promoter system should be tight in *E. coli* (Moncrief et al. 1997). Therefore, P_*tcdB*_ was inserted upstream of *pct*-*acrCBA* on pMTL83151_PA. Plasmid pMTL83151_PA was digested using *Eco*72I and *Sda*I and ligated with P_*tcdB*_ amplified from genomic DNA from *C. difficile* DSM 27147 using primers Inf_PtcdB_fw and Inf_PtcdB_rev to construct pMTL83151_P_*tcdB*__PA. The alternative sigma factor needed for P_*tcdB*_ recognition (TcdR; Martin-Verstraete et al. 2016) is provided on plasmid pMTL82251_LL and was added to the existing operon on this plasmid together with the lactose-inducible promoter P_*bgaL*_ from *C.* *perfringens* (Hartman et al. 2011) to control the expression of the PSO finally consisting of *ldhD*, *lcdC*, *lcdA*, *lcdB*, and *tcdR* (pMTL82251_P_*bgaL*__LL_tcdR). To construct pMTL82251_P_*bgaL*__LL_tcdR, plasmid pMTL82251_LL was linearized using *Xho*I and *Sda*I and fused with P_*tet*_ amplified from pMTL83151_P_*tet*__3-HP_opt using primers Inf_Ptet_fw and Inf_Ptet_LL_rev. Resulting plasmid pMTL82251_P_*tet*__LL was cut using *Nhe*I, and the generated backbone was used in a cloning reaction together with amplified *tcdR* (primers Inf_tcdR_wRBS_fw and Inf_tcdR_wRBS_rev; template genomic DNA from *C. difficile* DSM 27147) to build pMTL82251_P_*tet*__LL_tcdR. For construction of pMTL82251_P_*bgaL*__LL_tcdR, P_*bgaL*_ was amplified from plasmid pMTL83151_gusA_P_*bgaL*_ (Beck et al. 2020) using primers Inf_PbgaL_fw and Inf_PbgaL_rev and inserted in linearized pMTL82251_P_*tet*__LL_tcdR (linearized using enzymes *Xho*I and *Sal*I) to exchange P_*tet*_ against P_*bgaL*_. The resulting two-plasmid system is displayed in Figure S1.

Existing PSOs were reconstructed to even out the proposed imbalance in the acrylate pathway for optimization of propionate concentrations. Therefore, *pct* was moved to PSO2 (controlled by P_*bgaL*_ instead of P_*tcdB*_) whereas genes *lcdC*, *lcdA*, and *lcdB* were moved to the space formerly taken by *pct* in PSO1 (controlled by P_*tcdB*_ instead of P_*bgaL*_). Also, a second P_*tcdB*_ and a second *acr* gene cluster were inserted in PSO1 in two separate cloning steps (plasmids pMTL83151_P_*tcdB*__L_P_*tcdB*_ and pMTL83151_P_*tcdB*__L_P_*tcdB*__AA, respectively). Additionally, PSO2 was extended by another *tcdR* gene to yield pMTL82251_P_*bgaL*__LPTT. To construct the aforementioned plasmids, pMTL83151_P_*tcdB*__PA was cut using *Xba*I and *Nhe*I, and pMTL82251_P_*bgaL*__LL_tcdR was digested using *XmaJ*I and *Nhe*I. The genes and promoters to be relocated or inserted a second time were amplified using primers Inf_pct_fw and Inf_pct_rev (*pct*; template pMTL83151_P_*tcdB*__PA), Inf_lcd_fw and Inf_lcd_rev (*lcdCAB*; template pMTL82251_LL), Inf_tcdR_fw2 and Inf_tcdR_rev2 (*tcdR*; template pMTL82251_P_*bgaL*__LL_tcdR), as well as Inf_PtcdB_fw2 and Inf_PtcdB_rev2 (P_*tcdB*_; template pMTL83151_P_*tcdB*__PA) and fused with generated backbones to build plasmids pMTL83151_P_*tcdB*__L_P_*tcdB*_ and pMTL82251_P_*bgaL*__LPTT. In a second step, pMTL83151_P_*tcdB*__L_P_*tcdB*_ was linearized using enzymes *Xho*I and *Sgs*I and ligated with two *acr* gene clusters amplified from pMTL83151_P_*tcdB*__PA using primers Inf_acr_fw3 and Inf_acr_rev3 (*acr* cluster 1) as well as Inf_acr_fw4 and Inf_acr_rev4 (*acr* cluster 2) to construct pMTL83151_P_*tcdB*__L_P_*tcdB*__AA. The resulting plasmids are shown in Figure S2.

All plasmids constructed for the evaluation of promoter activities in *C. saccharoperbutylacetonicum* were based on the pMTL83251 backbone (Heap et al. 2009). Plasmid pMTL83251_P_*pta-ack*__FAST was kindly provided by Maximilian Flaiz (University of Ulm, unpublished). To construct further plasmids harboring *feg* (FAST-encoding gene) as a reporter gene, pMTL83251_P_*bgaL*__FAST (Flaiz et al. 2021) was linearized using *Bam*HI and *Nde*I and fused with PCR-amplified promoters P_*bld*_ (from *C. saccharoperbutylacetonicum*), P_*tcdB*_ (from *C. difficile*), and P_*lctB*_ (from *Acetobacterium woodii*). Templates for amplification of promoters were genomic DNA from *C. saccharoperbutylacetonicum* N1-4(HMT) DSM 14923 (P_*bld*_; primers Inf_Pbld_FAST_fw and Inf_Pbld_FAST_rev), plasmid pMTL83151_P_*tcdB*__PA (P_*tcdB*_; primers Inf_PtcdB_FAST_fw and Inf_PtcdB_FAST_rev), and genomic DNA from *A. woodii* DSM 1030 (P_*lctB*_; primers Inf_PlctB_FAST_fw and Inf_PlctB_FAST_rev). Final plasmids were pMTL83251_P_*bld*__FAST, pMTL83251_P_*tcdB*__FAST, and pMTL83251_P_*lctB*__FAST, respectively. To monitor the activity of P_*tcdB*_, plasmids carrying *feg* under control of P_*tcdB*_ and *tcdR* controlled by either P_*bld*_ or P_*bgaL*_ were constructed. For that purpose, pMTL83251_P_*tcdB*__FAST was digested using *Not*I and *Nde*I and ligated with fragments P_*bgaL*_ and *tcdR* (both amplified from pMTL82251_P_*bgaL*__LL_tcdR using primers Inf_PbgaL_tcdR-fdx_fw and Inf_PbgaL_tcdR-fdx_rev (P_*bgaL*_) as well as Inf_tcdR-fdx_fw and Inf_tcdR-fdx_rev (*tcdR*)) to yield pMTL83251_P_*tcdB*__FAST_P_*bgaL*__tcdR. Then, pMTL83251_P_*tcdB*__FAST_P_*bgaL*__tcdR was linearized with *Sma*I and *Sal*I and P_*bgaL*_ was exchanged against P_*bld*_ (amplified from pMTL83251_P_*bld*__FAST with primers Inf_Pbld_fw2 and Inf_Pbld_rev2) to assemble plasmid pMTL83251_P_*tcdB*__FAST_P_*bld*__tcdR.

### Transformation of bacterial strains

Preparation and transformation of chemically competent *E. coli* cells were performed following procedures previously described by Weitz et al. (2021). Transformation of *C. saccharoperbutylacetonicum* was performed using electroporation. Electrocompetent cells were prepared following known procedures (Wirth and Dürre 2021) with slight modifications. Cells were cultivated at 30 °C until mid-exponential growth phase (OD_600_ of 0.8–1.2). Centrifugation steps were performed in an anaerobic cabinet for 10 min at 3,985 × g and 4 °C. Recovery of pulsed cells was performed for 2–16 h at 32 °C.

### Analytical methods

The growth of bacterial strains was monitored by measuring the optical density at a wavelength of 600 nm (OD_600_) using the “Ultrospec™ 3100 pro UV/Visible” spectrophotometer (Amersham Biosciences Europe GmbH, Freiburg, Germany).

Quantification of metabolic products acetone, ethanol, propanol, butanol, acetate, propionate, and butyrate was achieved using gas chromatography (GC). In total, 2-ml samples were withdrawn from cultures throughout the course of growth experiments and centrifuged (15,000 × g, 30 min, 4 °C). A total of 480 µl of supernatant were filled into 2-ml crimp vials (CS-Chromatographie Service GmbH, Langerwehe, Germany), acidified by addition of 20 µl 2 M HCl, and closed with aluminum caps. Prepared samples were analyzed using a “Clarus 600” gas chromatograph (PerkinElmer, Inc., Waltham, MA, USA) equipped with an “Elite-FFAP” capillary column (inner diameter 0.32 mm × 30 m) and a flame ionization detector operating at 300 °C. H_2_ served as carrier gas with a flow rate of 2.25 ml min^−1^, injection temperature was set to 225 °C, detector gases were H_2_ (45 ml min^−1^) and synthetic air (79.5% N_2_ + 20.5% O_2_ at 450 ml min^−1^). 1 µl of culture supernatant was injected into the gas chromatograph and analyzed using the following temperature profile: 80 °C for 2 min followed by a gradual increase of temperature to 190 °C with a rate of 10 °C min^−1^, then, the temperature was increased to 250 °C at 40 °C min^−1^, and finally 250 °C were held constant for 1 min. For calibration purposes, defined external standards containing all substances were prepared.

Glucose consumption and lactate production were determined via high-performance liquid chromatography (HPLC) using the “Infinity 1260” HPLC system (Agilent Technologies, Santa Clara, CA, USA) equipped with a “CS-Organic-Acid Resin column” (150 × 8 mm; CS-Chromatographie Service GmbH, Langerwehe, Germany), a refraction index detector (for glucose) operating at 35 °C, and a diode array UV detector (for lactate) operating at a wavelength of 210 nm and room temperature. Culture supernatants were prepared as described for GC; however, no acidification of samples was necessary. As mobile phase, 5 mM H_2_SO_4_ with a flow rate of 0.7 ml min^−1^ was used. The sample volume injected into the HPLC system was 20 µl, and external standards again were prepared for calibration of determined compounds.

### FAST reporter assays

To monitor fluorescence levels of FAST-producing strains, 0.5–2-ml samples were withdrawn from cultures at different stages of growth and processed as described by Flaiz et al. (2021) with one exception. Washing steps were carried out for 10 min at 7,607 × g and 4 °C. To determine the fluorescence intensities (FLU) of FAST-producing cultures, a microplate reader was used. Fluorescence was determined following the established protocol of Flaiz et al. (2021) with one exception. Instead of 10 µM ^TF^Lime, only 5 µM ^TF^Lime were supplemented to PBS-suspended cells. FLU of cells without the addition of ^TF^Lime was determined as a negative control. Finally, fluorescence intensities were normalized to OD_600_ of PBS-suspended cells. Normalized FLU without the addition of ^TF^Lime were subtracted from FLU with ^TF^Lime to determine the actual FLU of the culture. Monitoring of FLU at single-cell level was performed via flow cytometry following procedures as described by Flaiz et al. (2022). Staining of washed cells was achieved by the addition of 5 µM ^TF^Lime (final OD_600_: 0.08).

## Results

### Construction of a two-plasmid system for propionate production

The construction of a PSO harboring all acrylate pathway genes and *ldhD* was not possible due to recurring mutations detected in the *acrC* gene upon inoculation of the respective *E. coli* strain from conserved stocks. Various cloning strategies using different promoters, origins of replication, and *E. coli* cloning hosts failed (data not shown). Therefore, the P_*tcdB*_-*tcdR* promoter system from *C. difficile* was chosen to tightly control the expression of the *acr* gene cluster. The P_*tcdB*_ promoter is dependent on induction with an alternative sigma factor (TcdR) as its -10 region does not contain the typical TATA motif and therefore cannot be recognized by standard bacterial sigma factors. Only when TcdR interacts with the RNA polymerase, transcription from P_*tcdB*_ can be initiated (Mani and Dupuy 2001; Martin-Verstraete et al. 2016). Thus, as long as TcdR is not provided in the same cell as P_*tcdB*_, expression of the genes under the control of P_*tcdB*_ should not be possible. To make use of this unique promoter system, the originally planned PSO was divided into two parts and assembled on a two-plasmid system (Fig. S1). PSO1 consisting of the *acr* gene cluster as well as the *pct* gene was put under control of P_*tcdB*_, whereas PSO2, which harbored genes *ldhD*, *lcdA*, *lcdB*, and *lcdC*, was extended by the *tcdR* gene. This way, leaky expression of *acrC* in *E. coli* could be prevented as no mutations were detected and cloning of both plasmids was successful (data not shown).

### Determination of promoter activities using FAST

**Fig. 2:**
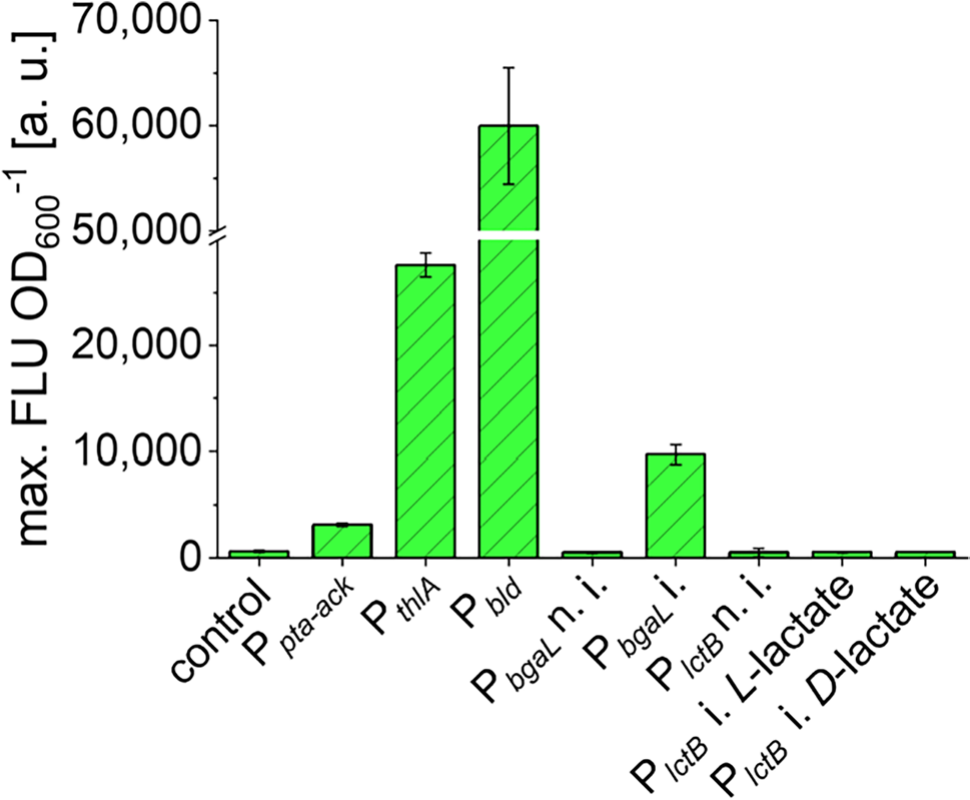
Maximal fluorescence intensities measured for FAST-producing *C. saccharoperbutylacetonicum* strains. All strains carried pMTL83251-based plasmids with *feg* under control of different promoters. Control, pMTL83251 without *feg*; P_*pta-ack*_, *feg* under control of P_*pta-ack*_ from *C. ljungdahlii*; P_*thlA*_, *feg* under control of P_*thlA*_ from *C. acetobutylicum*; P_*bld*_, *feg* under control of P_*bld*_ from *C. saccharoperbutylacetonicum*; P_*bgaL*_, *feg* under control of P_*bgaL*_ from *C. perfringens* (n. i., non-induced; i., induced using 20 mM lactose); P_*lctB*_, *feg* under control of P_*lctB*_ from *A. woodii* (n. i., non-induced; i., induced using 15 mM *D*- or *L*-lactate, respectively). Fluorescence intensities (FLU) were normalized to OD_600_ and are given in arbitrary units (a. u.). Error bars indicate standard deviations, *n* = 3

Since P_*tcdB*_ needs to be induced by the alternative sigma factor TcdR, the expression of *tcdR* has to be controlled separately. A promoter study using FAST as a fluorescent reporter was conducted to assess the strength and activity pattern of different clostridial promoters throughout the growth of *C. saccharoperbutylacetonicum*. This way, suitable promoters to drive *tcdR* expression should be identified. An overview of tested promoters as well as their known characteristics is given in Table 3. Figure 2 summarizes the maximal fluorescence intensities (FLU) detected for all promoters screened normalized to the OD_600_. The highest activity was measured for the promoter P_*bld*_ from *C. saccharoperbutylacetonicum* as the respective strain reached a FLU OD_600_^−1^ of 60,000. With this value, FLU of *C. saccharoperbutylacetonicum* [pMTL83251_P_*bld*__FAST] was more than twice as high as the FLU of *C. saccharoperbutylacetonicum* expressing *feg* under control of P_*thlA*_ from *C. acetobutylicum* (27,600) and approx. six-fold higher than FLU of the induced *C. saccharoperbutylacetonicum* [pMTL83251_P_*bgaL*__FAST] (9,500). The strain *C. saccharoperbutylacetonicum* [pMTL83251_P_*pta-ack*__FAST], which carried the P_*pta-ack*_ promoter from *C. ljungdahlii*, was only weakly fluorescent. Its maximal FLU value of 3,100 was the lowest FLU measured for any strain aside from the autofluorescence displayed by *C. saccharoperbutylacetonicum* strains without *feg* expression, i.e., *C. saccharoperbutylacetonicum* [pMTL83251] and non-induced *C. saccharoperbutylacetonicum* strains with *feg* under control of P_*bgaL*_ from *C. perfringens* or P_*lctB*_ from *A. woodii*, respectively. The strain *C. saccharoperbutylacetonicum* [pMTL83251_P_*lctB*__FAST] also only showed autofluorescence when induced with *D*- or *L*-lactate indicating that P_*lctB*_ is not active in *C. saccharoperbutylacetonicum*. Expression profiles of the strains revealed that all promoters have dynamic activities as FLU of all strains first rose during exponential growth phases and subsided with ongoing cultivation (Fig. S3). Only P_*pta-ack*_ showed a steady activity as FLU was constant between the mid-exponential and the mid-stationary phase. Furthermore, the two strongest promoters of this study, P_*bld*_ and P_*thlA*_, were confirmed to have an exponential growth phase-associated activity.

**Table 3:** Overview of promoters used for FAST reporter assay in *C. saccharoperbutylacetonicum*

Promoter	Origin	Features	Reference
P_*bgaL*_	*C. perfringens*	lactose-inducible	Hartman et al. 2011
P_*bld*_	*C. saccharoperbutylacetonicum*	exponential growth phase-associated activity	Kosaka et al. 2007
P_*lctB*_	*A. woodii*	lactate-inducible	Schölmerich et al. 2018
P_*pta-ack*_	*C. ljungdahlii*	constitutive	Hoffmeister et al. 2016
P_*thlA*_	*C. acetobutylicum*	early growth phase-associated activity (from early- to mid-exponential phase)	Tummala et al. 1999

**Fig. 3:**
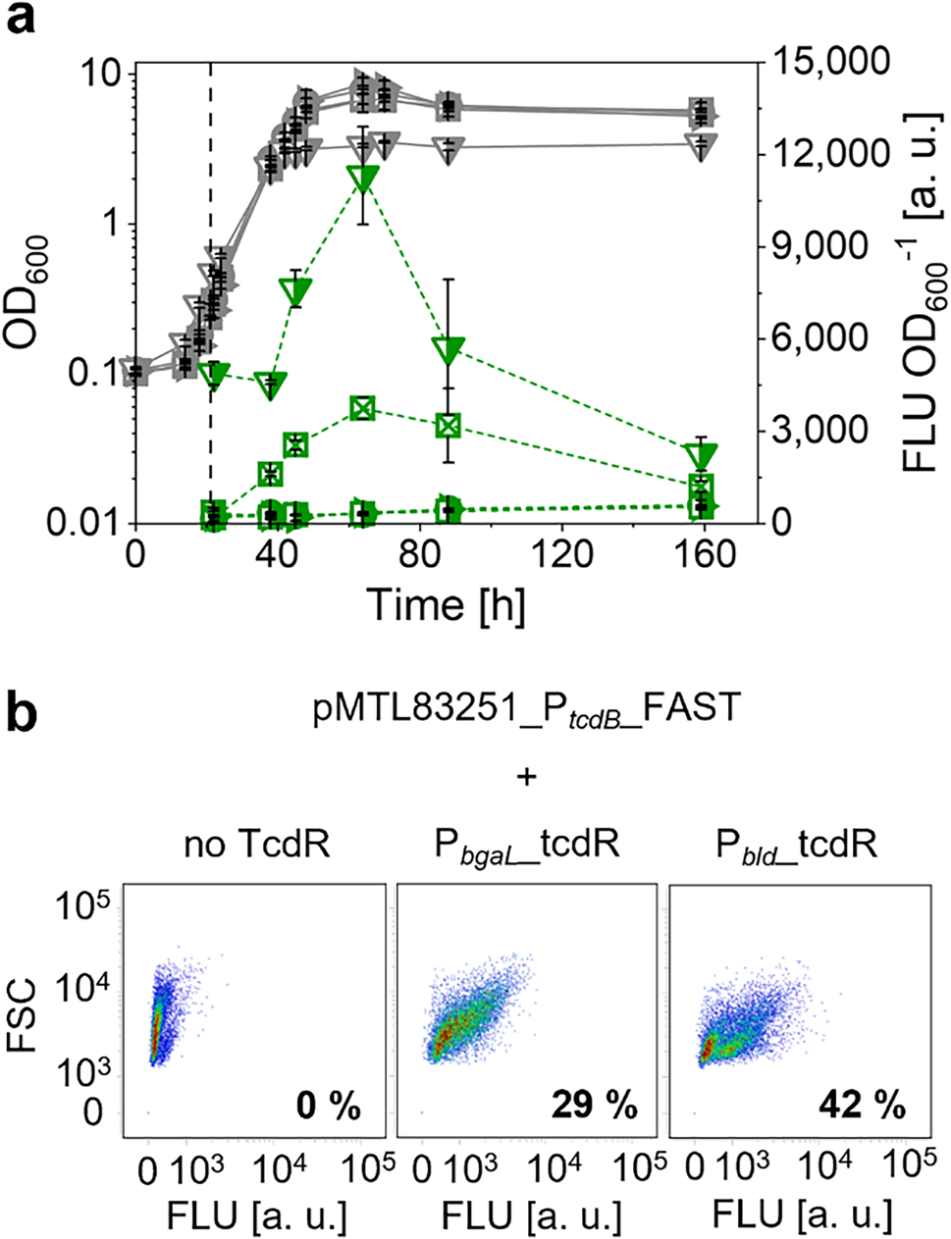
Evaluation of P_*tcdB*_ activity in *C. saccharoperbutylacetonicum* using FAST assay. All strains carried pMTL83251-based plasmids with *feg* under control of P_*tcdB*_ from *C. difficile* and *tcdR* under control of P_*bgaL*_ from *C. perfringens* or P_*bld*_ from *C. saccharoperbutylacetonicum*. **a** Growth (solid lines) and fluorescence intensities (dashed lines) of *C. saccharoperbutylacetonicum* [pMTL83251] (circles), *C. saccharoperbutylacetonicum* [pMTL83251_P_*tcdB*__FAST] (right-facing triangles), *C. saccharoperbutylacetonicum* [pMTL83251_P_*tcdB*__FAST_P_*bgaL*__tcdR] with (crossed squares) and without (half-filled squares) induction of gene expression, and *C. saccharoperbutylacetonicum* [pMTL83251_P_*tcdB*__FAST_P_*bld*__tcdR] (half-filled downward-facing triangles). **b** Density plots of *C. saccharoperbutylacetonicum* [pMTL83251_P_*tcdB*__FAST] (left), induced *C. saccharoperbutylacetonicum* [pMTL83251_P_*tcdB*__FAST_P_*bgaL*__tcdR] (middle), and *C. saccharoperbutylacetonicum* [pMTL83251_P_*tcdB*__FAST_P_*bld*__tcdR] (right) after 64 h of cultivation. Black dashed line indicates time of induction of P_*bgaL*_ using 20 mM lactose. Error bars indicate standard deviations, *n* = 3

In a second step, the activity of P_*tcdB*_ was investigated in dependence on different levels of *tcdR* expression. For that purpose, recombinant *C. saccharoperbutylacetonicum* strains carrying plasmids with *feg* under the control of P_*tcdB*_ and *tcdR* under the control of either P_*bld*_ or P_*bgaL*_ were constructed. Promoters P_*bld*_ and P_*bgaL*_ were chosen due to the bright fluorescence of respective strains. Also, P_*bgaL*_ tightly regulated gene expression as FLU was only detected in the induced culture (Fig. 2). Subsequently, P_*tcdB*_ activity was investigated at both whole-culture and single-cell level using a microplate reader and flow cytometry. As negative controls, strains harboring the empty vector pMTL83251 or a plasmid with *feg* under control of P_*tcdB*_ but without a *tcdR* gene (pMTL83251_P_*tcdB*__FAST) were cultivated in parallel to strains *C. saccharoperbutylacetonicum* [pMTL83251_P_*tcdB*__FAST_P_*bld*__tcdR] and *C. saccharoperbutylacetonicum* [pMTL83251_P_*tcdB*__FAST_P_*bgaL*__tcdR]. As previously observed, FLU of FAST-producing strains was highly dynamic during growth of *C. saccharoperbutylacetonicum* (Fig. 3a). While the negative controls only showed autofluorescence, FLU of *C. saccharoperbutylacetonicum* [pMTL83251_P_*tcdB*__FAST_P_*bld*__tcdR] and induced *C. saccharoperbutylacetonicum* [pMTL83251_P_*tcdB*__FAST_P_*bgaL*__tcdR] increased during exponential growth phases and reached a maximum during early stationary phases. The maximum FLU of the strain with *tcdR* controlled by P_*bld*_ was approx. 28-fold higher than the autofluorescence of the negative controls and three-fold higher than FLU of the strain with lactose-inducible *tcdR* expression. With ongoing cultivation, FLU of both strains declined to levels comparable to empty vector and non-induced strains. Flow cytometric analyses of the FAST-producing strains revealed heterogenous populations of fluorescent and non-fluorescent *C. saccharoperbutylacetonicum* cells (Fig. 3b). While *C. saccharoperbutylacetonicum* [pMTL83251_P_*tcdB*__FAST] did not show any fluorescence besides the previously described autofluorescence, induction of *tcdR* expression with lactose resulted in a shift of the population, indicating fluorescence due to an activated P_*tcdB*_. Similarly, cells with autonomous *tcdR* expression (P_*bld*_ control), and thus, autonomic P_*tcdB*_ induction became fluorescent as the population shifted in direction of increasing FLU. After 64 h of cultivation, the maximal amounts of fluorescent cells were detected for both FAST-producing cultures, which accounted for 29% and 42% for P_*bgaL*_- and P_*bld*_-controlled *tcdR* expression, respectively. Additional data on flow cytometric analyses of the strains including the investigation of FAST production in strains *C. saccharoperbutylacetonicum* [pMTL83251_P_*bld*__FAST] and *C. saccharoperbutylacetonicum* [pMTL83251_P_*bgaL*__FAST] are shown in Figure S4. When *feg* expression was directly controlled by P_*bld*_ or P_*bgaL*_, the maximum amounts of fluorescent cells were significantly higher compared to *feg* expression controlled by P_*tcdB*_ resulting in a total of 97% and 76% of fluorescent cells, respectively. In later stages of growth, FLU of all strains decreased as populations became non-fluorescent. Although P_*tcdB*_ activity was higher when *tcdR* expression was under control of P_*bld*_, the lactose-inducible P_*bgaL*_ promoter was chosen to establish propionate production in *C. saccharoperbutylacetonicum* to avoid possible pathway overloading due to the high activity observed for P_*bld*_ (Grosse-Honebrink et al. 2021). Furthermore, the use of P_*bgaL*_ allows a controlled induction of gene expression and has recently successfully been used to establish a two-plasmid system carrying P_*tcdB*_ and *tcdR* in *C. saccharoperbutylacetonicum* (Flaiz et al. 2022).

#### Establishment of propionate production in *C. saccharoperbutylacetonicum*

**Fig. 4:**
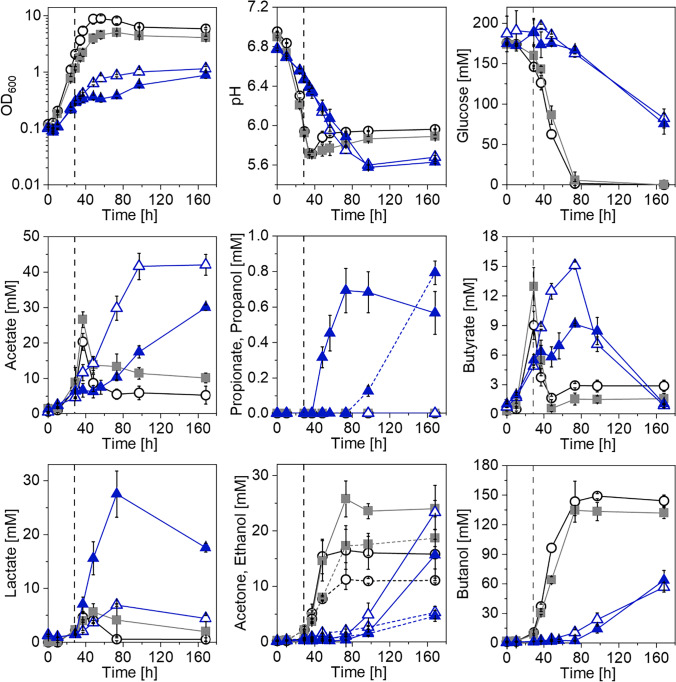
Results of growth experiment to establish propionate production in *C. saccharoperbutylacetonicum*. Growth (OD_600_), pH, glucose consumption, and product formation were monitored throughout the course of the experiment. Open circles, *C. saccharoperbutylacetonicum* wild type; squares, *C. saccharoperbutylacetonicum* [pMTL83151][pMTL82251]; open triangles, non-induced *C. saccharoperbutylacetonicum* [pMTL83151_P_*tcdB*__PA][pMTL82251_P_*bgaL*__LL_tcdR]; filled triangles, induced *C. saccharoperbutylacetonicum* [pMTL83151_P_*tcdB*__PA][pMTL82251_P_*bgaL*__LL_tcdR]. Induction of gene expression was achieved by addition of 20 mM lactose and is indicated with a black dashed line. Dotted lines indicate ethanol or propanol production of respective strains. Error bars represent standard deviations, *n* = 3

As previously mentioned, a two-plasmid system carrying the two PSOs was constructed to convert *C. saccharoperbutylacetonicum* into a propionate producer (Fig. S1). These two plasmids were used to transform *C. saccharoperbutylacetonicum*, resulting in the strain *C. saccharoperbutylacetonicum* [pMTL83151_P_*tcdB*__PA][pMTL82251_P_*bgaL*__LL_tcdR]. As a negative control, a strain carrying the empty vectors pMTL83151 and pMTL82251 was constructed. Recombinant strains were then tested in comparison to *C. saccharoperbutylacetonicum* wild type in minimal medium using glucose as a carbon source (Fig. 4). The strain harboring the PSOs showed considerable differences in growth and production behavior compared to the control strains. Aside from a strongly diminished growth and incomplete glucose consumption, acetate was accumulated in both induced and non-induced *C. saccharoperbutylacetonicum* [pMTL83151_P_*tcdB*__PA][pMTL82251_P_*bgaL*__LL_tcdR] cultures (30 mM and 42.1 mM). Butyrate formation of *C. saccharoperbutylacetonicum* strains carrying PSOs was comparable to the control strains; however, reassimilation of this acid was delayed by 44 h. The induced culture of *C. saccharoperbutylacetonicum* [pMTL83151_P_*tcdB*__PA][pMTL82251_P_*bgaL*__LL_tcdR] produced 27.5 mM lactate, 0.7 mM propionate, and 0.8 mM propanol, whereas the controls and the non-induced strain only produced little lactate (4.7 to 6.9 mM) and no propionate or propanol. This provided evidence for the first heterologous production of propionate achieved in any *Clostridium* sp. Solvent production of *C. saccharoperbutylacetonicum* strains carrying the PSOs was delayed and reduced compared to the control strains, which is also reflected in the pH development. While the pH of *C. saccharoperbutylacetonicum* wild type and the vector control strain increased to values of 6 and 5.9 after a drop during the first 37 h of cultivation, pH of *C. saccharoperbutylacetonicum* [pMTL83151_P_*tcdB*__PA][pMTL82251_P_*bgaL*__LL_tcdR] dropped to a value of 5.6 and only slightly increased after 97 h. Final acetone titers of non-induced and induced *C. saccharoperbutylacetonicum* [pMTL83151_P_*tcdB*__PA][pMTL82251_P_*bgaL*__LL_tcdR] were in the range of the vector control and wild type strains, respectively. However, only half the amount of ethanol and butanol were produced by the PSO-carrying strains.

#### Optimization of propionate production in *C. saccharoperbutylacetonicum*

**Fig. 5:**
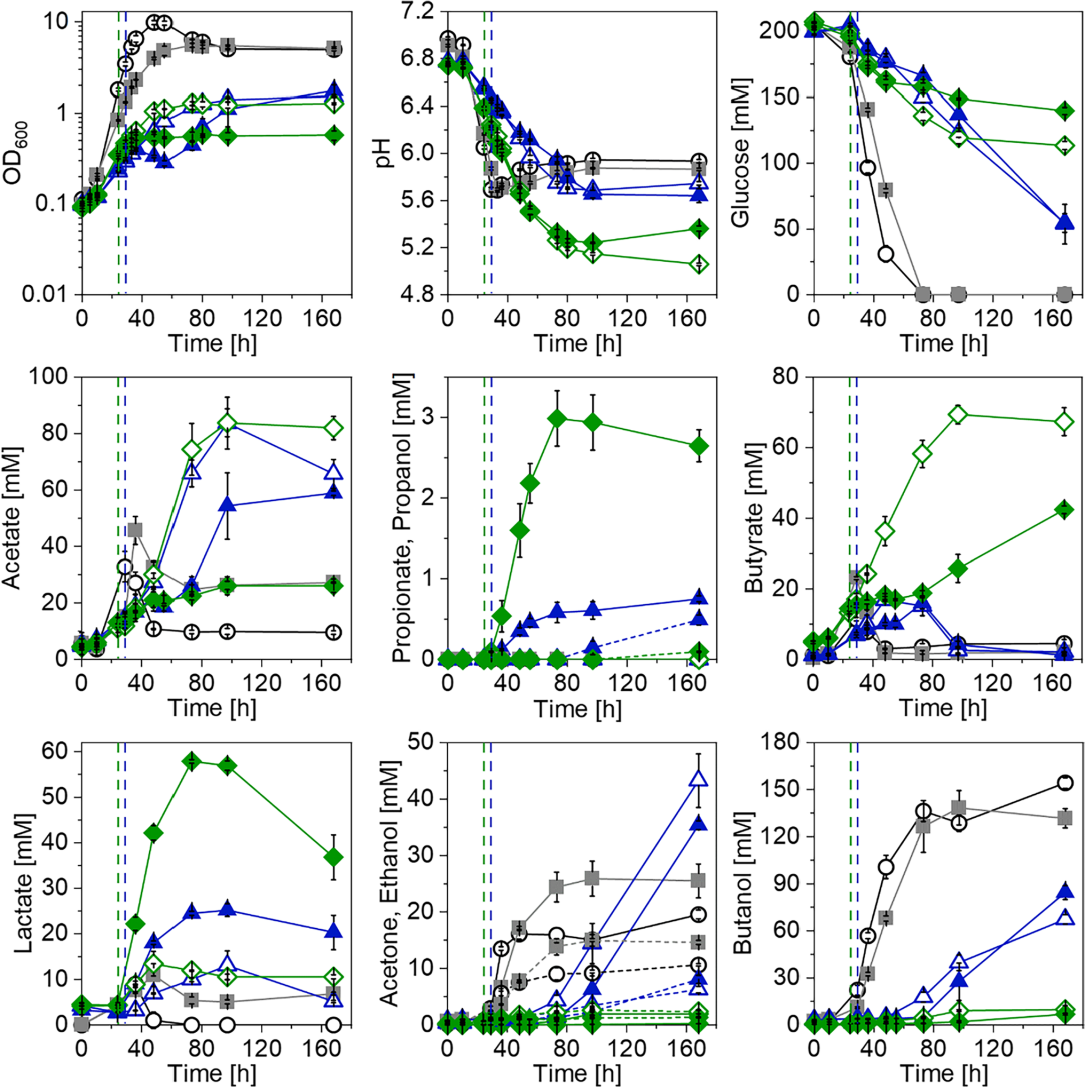
Results of growth experiment to improve propionate production in *C. saccharoperbutylacetonicum*. Growth (OD_600_), pH, glucose consumption, and product formation were monitored throughout the course of the experiment. Open circles, *C. saccharoperbutylacetonicum* wild type; squares, *C. saccharoperbutylacetonicum* [pMTL83151][pMTL82251]; open triangles, non-induced *C. saccharoperbutylacetonicum* [pMTL83151_P_*tcdB*__PA][pMTL82251_P_*bgaL*__LL_tcdR]; filled triangles, induced *C. saccharoperbutylacetonicum* [pMTL83151_P_*tcdB*__PA][pMTL82251_P_*bgaL*__LL_tcdR]; open diamonds, non-induced *C. saccharoperbutylacetonicum* [pMTL83151_P_*tcdB*__L_P_*tcdB*__AA][pMTL82251_P_*bgaL*__LPTT]; filled diamonds, induced *C. saccharoperbutylacetonicum* [pMTL83151_P_*tcdB*__L_P_*tcdB*__AA][pMTL82251_P_*bgaL*__LPTT]. Induction of gene expression was achieved by addition of 20 mM lactose and is indicated with dashed lines. Dotted lines indicate ethanol or propanol production of respective strains. Error bars represent standard deviations, *n* = 3

The productivity of the recombinant strain *C. saccharoperbutylacetonicum* [pMTL83151_P_*tcdB*__PA][pMTL82251_P_*bgaL*__LL_tcdR] with an induced gene expression was rather low as only 0.7 mM propionate was detected (Fig. 4). Since the two PSOs were under the control of two promoters with different strengths (P_*tcdB*_ weaker than P_*bgaL*_), a metabolic imbalance was proposed as the reason for the low productivity. To even out this postulated imbalance and ensure a more balanced carbon flux through the recombinant pathway, the two PSOs were reconstructed. The resulting new plasmid system carried two copies of the *acr* and one copy of the *lcd* gene cluster under the control of separate P_*tcdB*_, as well as the *pct*, the *ldhD*, and two *tcdR* genes under the control of P_*bgaL*_ (Fig. S2). The new PSOs were used to transform *C. saccharoperbutylacetonicum* to build the strain *C. saccharoperbutylacetonicum* [pMTL83151_P_*tcdB*__L_P_*tcdB*__AA][pMTL82251_P_*bgaL*__LPTT]. This strain was then characterized in a growth experiment in comparison to the original producer *C. saccharoperbutylacetonicum* [pMTL83151_P_*tcdB*__PA][pMTL82251_P_*bgaL*__LL_tcdR] as well as the negative controls *C. saccharoperbutylacetonicum* [pMTL83151][pMTL82251] and *C. saccharoperbutylacetonicum* wild type (Fig. 5). Recombinant strains carrying old and new PSOs showed impaired growth and did not deplete the provided glucose. The induced strain carrying the optimized PSOs produced up to 26.1 mM acetate, 42.4 mM butyrate, 57.9 mM lactate, and 3 mM propionate. Solvent production was limited to traces of acetone (0.2 mM) and propanol (0.1 mM) as well as small amounts of ethanol (1.3 mM) and butanol (6.8 mM). Aside from a lactate uptake, no acid reassimilation was observed for this strain. The non-induced *C. saccharoperbutylacetonicum* [pMTL83151_P_*tcdB*__L_P_*tcdB*__AA][pMTL82251_P_*bgaL*__LPTT] also accumulated acids rather than solvents; however, this strain produced less lactate and more acetate and butyrate. *C. saccharoperbutylacetonicum* harboring the original PSOs showed a growth and production profile similar to the growth experiment displayed in Fig. 4. Again, acetate was accumulated, and butyrate was reassimilated with a delay compared to the control strains. Lactate production of the induced *C. saccharoperbutylacetonicum* [pMTL83151_P_*tcdB*__PA][pMTL82251_P_*bgaL*__LL_tcdR] strain was elevated (25.2 mM), propionate concentration reached a maximum of 0.8 mM. Moreover, 0.5 mM propanol was detected. Further solvents produced by non-induced and induced *C. saccharoperbutylacetonicum* [pMTL83151_P_*tcdB*__PA][pMTL82251_P_*bgaL*__LL_tcdR] were acetone (up to 43.3 mM), ethanol (up to 8.1 mM), and butanol (up to 84.5 mM). A direct comparison of the original and the optimized *C. saccharoperbutylacetonicum* strains revealed an increase in propionate production of 375%. Thus, the reconstruction of the PSOs led to an almost four-fold increase in propionate.

## Discussion

The data presented clearly show that heterologous expression of the acrylate pathway leads to propionate production in *C. saccharoperbutylacetonicum*. In the first growth experiment, only traces of propionate (0.7 mM) were obtained, indicating that the carbon flux through the established pathway was not high. Although induction of gene expression led to elevated lactate concentrations in comparison to control strains, possibly a result of the heterologously expressed *D*-lactate dehydrogenase, lactate turnover to propionate seemed to be limited. The acrylate pathway consists of multiple steps starting with the activation of *D*-lactate to its CoA-derivative *D*-lactoyl-CoA, which is subsequently dehydrated to the toxic compound acryloyl-CoA. These reactions are catalyzed by the propionate CoA-transferase and lactoyl-CoA dehydratase, respectively. The resulting acryloyl-CoA is reduced to propionyl-CoA by an acryloyl-CoA reductase. Finally, propionate is released as the product by a CoA transfer from propionyl-CoA to *D*-lactate by the Pct (Fig. 1; Hetzel et al. 2003). Considering the fact that the PSOs encoding all mentioned enzymes were controlled by two different promoters, which FAST studies showed to be highly different in strength (Figs. 2, 3, and S4), the concern of a metabolic imbalance is valid. Lcd-encoding genes were under the control of the stronger P_*bgaL*_ promoter, thus possibly leading to a higher expression level compared to the *acr* genes, which were controlled by the weaker P_*tcdB*_ promoter. Based on these assumptions it would follow that the Lcd turnover is higher than the Acr turnover, thereby leading to an acryloyl-CoA accumulation. Furthermore, the Acr apparently has a low catalytic efficiency, which, according to reports is so low that native producers must compensate for this by producing high amounts of the enzyme (Hetzel et al. 2003; Kandasamy et al. 2013). Therefore, it is likely that acryloyl-CoA was accumulated rather than converted by Lcd and Acr reactions. Due to the electrophilic properties of acryloyl-CoA (Herrmann et al. 2005), it seems logical that an accumulation of this toxic compound would cause a high stress level for the production host, which could manifest itself in an impaired growth, a delayed or complete lack of acid reassimilation and solventogenesis, and a low productivity, all of which was observed in the growth experiments conducted in this study. Also, since the acrylate pathway is an electron sink, a deficiency in reducing equivalents could be a consequence (Kandasamy et al. 2013). With the metabolism out of balance due to the high burden exerted by the accumulation of pathway intermediates such as acryloyl-CoA and depletion of the NADH pool, high strain performances cannot be expected. Furthermore, FAST studies conducted via flow cytometry revealed that *C. saccharoperbutylacetonicum* cultures were heterogeneous even in the case of *feg* expression mediated by the strong and native P_*bld*_ promoter (Figs. 3 and S4). Especially the use of the P_*tcdB*_-*tcdR* system with *tcdR* expression driven by P_*bgaL*_, which reflects the situation created in the propionate-producing strains, seems to limit the amount of productive cells as only 29% showed fluorescence in presence of ^TF^Lime (Fig. 3b). When transferring this observation to the strains carrying PSOs under control of P_*tcdB*_, it is reasonable to assume that only a small portion of the culture expressed the acrylate pathway genes and thus was able to form propionate. Such heterogeneity of bacterial populations in connection with an induced, plasmid-based gene expression has been shown several times and hypothesized to be caused by the uptake mechanism and concentration of the inducer, plasmid instability, or plasmid loss (Binder et al. 2016; Flaiz et al. 2021; Siegele and Hu 1997). Further factors to contribute to culture heterogeneity are cell morphogenesis or sporulation, both of which are especially apparent in clostridial cultures (Jones et al. 2008; Tracy et al. 2010). Most recently, Flaiz et al. (2022) also demonstrated heterogeneity of *C. saccharoperbutylacetonicum* cultures expressing *feg* in a P_*tcdB*_-dependent manner with *tcdR* controlled by P_*bgaL*_. Again, culture heterogeneity was observed throughout all growth phases, although in this case, the number of fluorescent cells was higher compared to the present study (Flaiz et al. 2022). Nevertheless, the impact of culture heterogeneity on the production behavior of *C. saccharoperbutylacetonicum* cannot be neglected and has to be considered a major contributor to the low observed productivity aside from the postulated metabolic imbalance.

The rearrangement of acrylate pathway genes to overcome the postulated metabolic imbalance and circumvent the bottleneck created by the Acr led to an increase in propionate production by almost four-fold. Interestingly, introduction of the optimized PSOs led to a shift of metabolic products from solvents to acids. While the non-induced *C. saccharoperbutylacetonicum* strain accumulated 83.8 mM acetate and 69.4 mM butyrate, the induced strain first produced 57.9 mM lactate, which was partially reassimilated and used for butyrate and propionate formation. The reason for this shift is not completely clear, however, can possibly be explained by the increased demand of the strain for ATP to maintain and express the enlarged PSOs. Both acetate and butyrate formation are important energy sources as they involve the formation of one ATP via substrate-level phosphorylation (Boynton et al. 1996; Hartmanis 1987), thus increasing the amount of available ATP. With an increased turnover of glucose to acids, the demand for reducing equivalents, especially oxidized ferredoxin but also NADH, increases in parallel as these are needed by the pyruvate:ferredoxin oxidoreductase, 3-hydroxybutyryl-CoA dehydrogenase, and butyryl-CoA dehydrogenase reactions (Jones and Woods 1986). In addition, the acrylate pathway consumes NADH during several steps. This raises the question of how the strain can adapt its metabolism to meet this increased need for reducing power. Under normal conditions, carbon flow from glucose to each of the acids and solvents happens in a particular ratio so that carbon and redox balances are closed (Jones and Woods 1986). However, solventogenesis is a process involving a high turnover of NADH to form ethanol and butanol (Jones and Woods 1986). Thus, a reduction of solventogenesis could save a substantial amount of NADH to be invested in acidogenesis including propionate formation via the acrylate pathway. Since *C. saccharoperbutylacetonicum* strains harboring optimized PSOs only produced little solvents (Fig. 5), it seems as if solventogenesis was indeed spared for the benefit of the energetically more favorable acidogenic pathways. To meet the increased need for oxidized ferredoxin, the strain can use its Rnf complex to recover oxidized ferredoxin and form NADH, which is accompanied by the generation of an ion gradient (Poehlein et al. 2017). This in turn can be used by the ATPase for further ATP formation and to fill up the ATP pool.

Although propionate production was successfully increased, the overall titer of 3 mM is still low. Considering the partial propionate reassimilation observed in both growth experiments, propionate titers could have been higher had it not been reduced to propanol. The formation of propanol is most probably due to the uptake of propionate by the acetoacetyl-CoA:acetate/butyrate CoA-transferase, which has a broad substrate spectrum including propionate (Hartmanis et al. 1984). The resulting propionyl-CoA can then be converted to propanol by aldehyde and alcohol dehydrogenases. Aside from propanol, other by-products such as solvents and butyrate limit the level of produced propionate as their formation requires both carbon and reducing equivalents. Other studies targeting heterologous propionate production using different host strains also reported challenges leading to mixed results. While *E. coli* engineered with the Sleeping beauty mutase operon and carrying multiple gene deletions or recombinant *Ps. putida* cultivated in fed-batch mode achieved a maximum of approx. 160 and 823 mM propionate from 326 mM glycerol and 850 mM *L*-threonine, respectively (Akawi et al. 2015; Mu et al. 2021), bacterial strains modified with the acrylate pathway also only produced rather low amounts of propionate. These ranged between 0.01 mM for *L. plantarum* (Balasubramanian and Subramanian 2019) and 3.7 mM for *E. coli* (Kandasamy et al. 2013), the latter of which is comparable to the propionate concentration produced by *C. saccharoperbutylacetonicum* [pMTL83151_P_*tcdB*__L_P_*tcdB*__AA][pMTL82251_P_*bgaL*__LPTT]. The low propionate titers could be due to metabolic imbalances leading to the accumulation of pathway intermediates, redox deficiencies, and low activities of recombinant enzymes as previously hypothesized by other groups (Balasubramanian and Subramanian 2019; Kandasamy et al. 2013). Despite these hurdles, there are options that might lead to an improved propionate production using *C. saccharoperbutylacetonicum* through which it could possibly outperform recombinant *E. coli* and *Ps. putida* strains or at least reach the same production level. One such option is the introduction of PSOs into strains harboring tailored mutations to improve carbon and redox balances. Since carbon and redox equivalents are predominantly invested in C_4_-producing pathways in *C. saccharoperbutylacetonicum*, manipulations in these metabolic branches might be promising. A deletion of 3-hydroxybutyryl-CoA dehydrogenase, crotonase, or aldehyde and alcohol dehydrogenases in either *C. saccharoperbutylacetonicum* or its close relative *C. acetobutylicum* led to a reduction or complete abolishment of butyrate or solvent formation (Baur 2022; Cooksley et al. 2012; Lehmann and Lütke-Eversloh 2011). Simultaneously, saved reducing equivalents were used for lactate or ethanol formation (Baur 2022; Lehmann and Lütke-Eversloh 2011). If such a strain carried the reductive acrylate pathway, this might lead to increased propionate concentrations. Other options for modifications to manipulate the carbon and electron flow in favor of the acrylate pathway would be the deletion of the hydrogenase gene cluster or the global regulator Spo0A. The ferredoxin hydrogenase of *C. saccharoperbutylacetonicum* produces hydrogen while simultaneously oxidizing reduced ferredoxin (Dada et al. 2013). Thus, a deletion of the hydrogenase could save reduced ferredoxin to be turned over by the Rnf complex, which as mentioned before, can use this for NADH generation. Deletion of *spo0A* was shown to reduce both solventogenesis and sporulation in many clostridial strains including *C. saccharoperbutylacetonicum* (Atmadjaja et al. 2019; Harris et al. 2002; Schwarz et al. 2017). Since sporulation is also a major contributor to culture heterogeneity (Tracy et al. 2010), a combination of *spo0A* deletion with the PSOs could result in increased propionate titers. Another option to overcome culture heterogeneity would be chromosomal integration of the PSOs as this would lead to a plasmid-independent expression and thereby eliminate plasmid loss or instability as possible limiting factors. Whether such an optimized *C. saccharoperbutylacetonicum* strain could then outperform native propionate producers such as propionibacteria remains questionable as even higher propionate titers were achieved by the cultivation of these bacteria in bioreactors using more sophisticated approaches that thus far have never been employed using *C. saccharoperbutylacetonicum*. The highest ever reported propionate concentrations are 1 M using *P. acidipropionici* (Liu et al. 2016) and 1.2 to 1.8 M using *P. freudenreichii* (Chen et al. 2013; Feng et al. 2011), when strains were cultivated in fed-batch mode with high cell density or immobilized cells and glucose or hydrolyzed sugar cane molasses as substrates. These concentrations were achieved with productivities of 4.3 to 7.7 mM h^−1^, which is 54- to 96-fold higher than the productivity of *C. saccharoperbutylacetonicum* [pMTL83151_P_*tcdB*__L_P_*tcdB*__AA][pMTL82251_P_*bgaL*__LPTT] (0.08 mM h^−1^). Nevertheless, it is imaginable that fed-batch or continuous cultivation of further optimized *C. saccharoperbutylacetonicum* could lead to another increase in propionate concentration and thus make it a strain that can very well compete but probably not outperform native producers with respect to the propionate concentration and productivity. Overall, *C. saccharoperbutylacetonicum* was successfully engineered as a propionate producer. Although propionate titer was rather low with 3 mM, different options for further strain engineering and cultivation are conceivable to increase the performance of recombinant *C. saccharoperbutylacetonicum* strains. Successfully engineered and improved strains could then possibly be considered for commercial propionate production using sustainable resources such as lignocellulosic hydrolysates.

## Supplementary information

Below is the link to the electronic supplementary material.

Supplementary file1 (PDF 1.13 MB)

## Data Availability

The datasets generated during and/or analyzed during the current study are available from the corresponding author upon reasonable request.
