# Supplementary material for: Production of propionate using metabolically engineered strains of Clostridium saccharoperbutylacetonicum
Source: Appl Microbiol Biotechnol. 2022 Oct 25;106(22):7547–62. doi: 10.1007/s00253-022-12210-8 (PMC9666320; doi:10.1007/s00253-022-12210-8)
Supplement: Supplementary file 1 — Supplementary file1 (PDF 1.13 MB) [file 253_2022_12210_MOESM1_ESM.pdf]

Supplementary material

Applied Microbiology and Biotechnology

Production of propionate using metabolically engineered strains of *Clostridium saccharoperbutylacetonicum*

Authors

Tina Baur[1*], Alexander Wentzel[2], Peter Dürre[1]

[1]University of Ulm, Institute of Microbiology and Biotechnology, Albert-Einstein-Allee 11, 89081 Ulm, Germany

[2]SINTEF Industry, Department of Biotechnology and Nanomedicine, Richard Birkelands vei 3, 7034 Trondheim, Norway

[*]Corresponding author: Tina Baur

e-mail address: tina.baur@uni-ulm.de

telephone: (0049)731-50-22713

fax number: (0049)731-50-22719

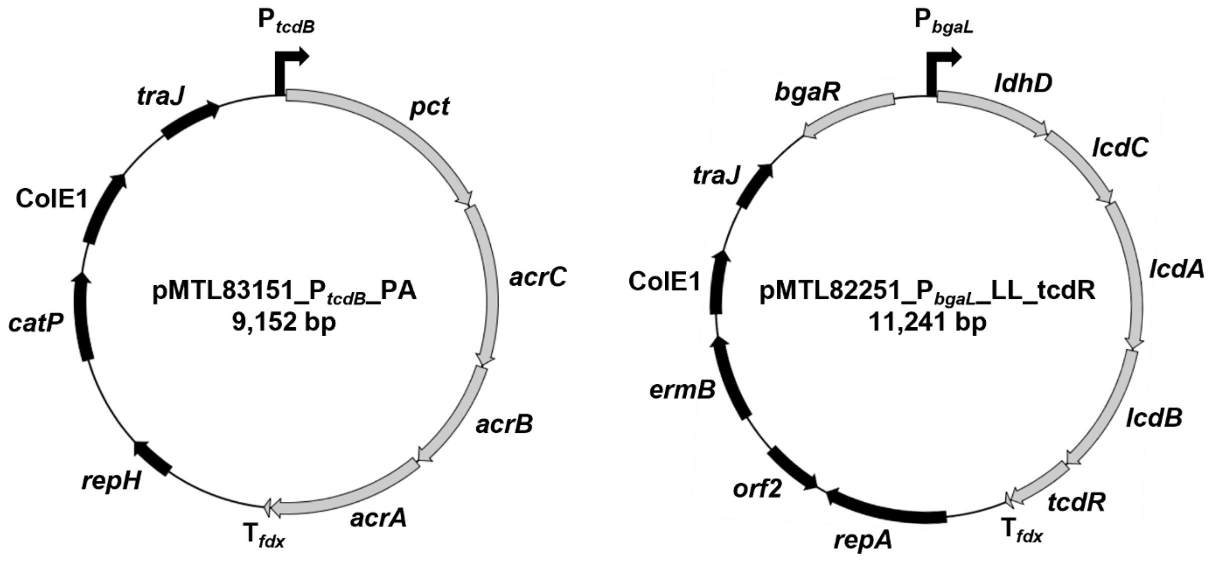

Fig. S1 Two-plasmid system to establish propionate production in *C. saccharoperbutylacetonicum*. All strains finally carried a combination of pMTL83151_P_{tcdB}_PA (left) and pMTL82251_P_{bgaL}_LL_tcdR (right). *repH*, Gram-positive replicon of pCB102 from *Clostridium butyricum*; *catP*, gene encoding chloramphenicol resistance; ColE1, Gram-negative replicon of pMTL20; *traJ*, gene encoding conjugal transfer function; *repA/orf2*, Gram-positive replicon of pBP1 from *Clostridium botulinum*; *ermB*, gene encoding erythromycin resistance; P_{tcdB}, promoter P_{tcdB} from *C. difficile*; *pct*, propionate CoA-transferase from *An. neopropionicum*; *acrC*, acryloyl-CoA reductase dehydrogenase (α) subunit from *An. neopropionicum*; *acrB*, acryloyl-CoA reductase electron-transferring flavoprotein (ETF)-β subunit from *An. neopropionicum*; *acrA*, acryloyl-CoA reductase ETF-γ subunit from *An. neopropionicum*; *bgaR*, gene encoding BgaR activator from *C. perfingens*; P_{bgaL}, lactose-inducible promoter P_{bgaL} from *C. perfringens*; *ldhD*, *D*-lactate dehydrogenase from *L. mesenteroides* subsp. *mesenteroides*; *lcdC*, lactyl-CoA dehydratase EI subunit from *An. neopropionicum*; *lcdA*, lactyl-CoA dehydratase EII-α subunit from *An. neopropionicum*; *lcdB*, lactyl-CoA dehydratase EII-β subunit from *An. neopropionicum*; *tcdR*, alternative sigma factor TcdR from *C. difficile*; T_{fdx}, terminator derived from *fdx* gene from *Clostridium pasteurianum*

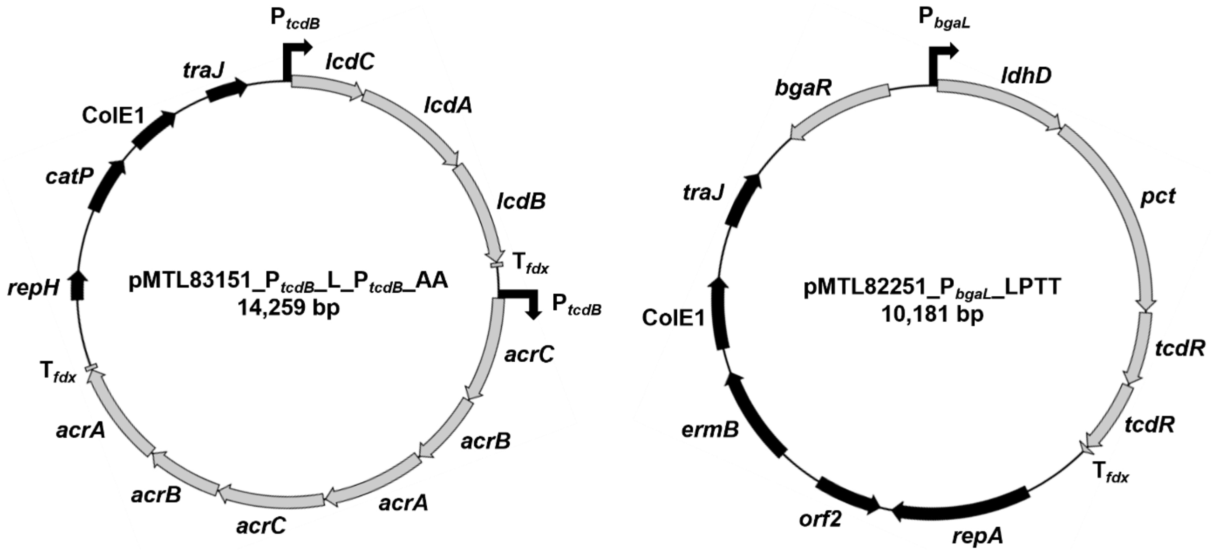

Fig. S2 Rearranged two-plasmid system to improve propionate production in *C. saccharoperbutylacetonicum*. All strains finally carried a combination of pMTL83151_P_{tcdB}_L_P_{tcdB}_AA (left) and pMTL82251_P_{bgaL}_LPTT (right). *repH*, Gram-positive replicon of pCB102 from *C. butyricum*; *catP*, gene encoding chloramphenicol resistance; ColE1, Gram-negative replicon of pMTL20; *traJ*, gene encoding conjugal transfer function; *repA/orf2*, Gram-positive replicon of pBP1 from *C. botulinum*; *ermB*, gene encoding erythromycin resistance; P_{tcdB}, promoter P_{tcdB} from *C. difficile*; *lcdC*, lactyl-CoA dehydratase EI subunit from *An. neopropionicum*; *lcdA*, lactyl-CoA dehydratase EII-α subunit from *An. neopropionicum*; *lcdB*, lactyl-CoA dehydratase EII-β subunit from *An. neopropionicum*; *acrC*, acryloyl-CoA reductase dehydrogenase (α) subunit from *An. neopropionicum*; *acrB*, acryloyl-CoA reductase electron-transferring flavoprotein (ETF)-β subunit from *An. neopropionicum*; *acrA*, acryloyl-CoA reductase ETF-γ subunit from *An. neopropionicum*; *bgaR*, gene encoding BgaR activator from *C. perfingens*; P_{bgaL}, lactose-inducible promoter P_{bgaL} from *C. perfringens*; *ldhD*, *D*-lactate dehydrogenase from *L. mesenteroides* subsp. *mesenteroides*; *pct*, propionate CoA-transferase from *An. neopropionicum*; *tcdR*, alternative sigma factor TcdR from *C. difficile*; T_{fdx}, terminator derived from *fdx* gene from *C. pasteurianum*

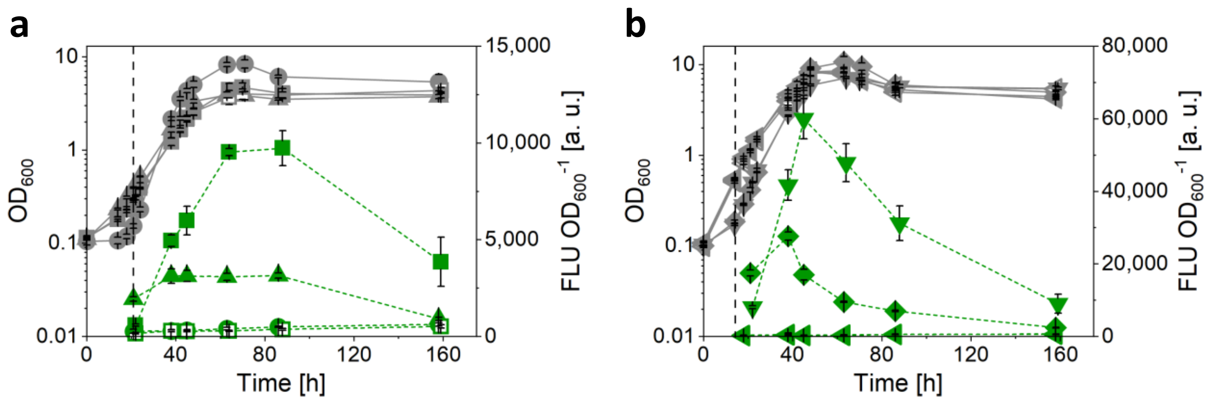

Fig. S3 Evaluation of promoter activities in *C. saccharoperbutylacetonicum* using FAST assay. All strains carried pMTL83251-based plasmids with *feg* under control of promoters with different origins. **a** growth (solid lines) and fluorescence intensities (dotted lines) of *C. saccharoperbutylacetonicum* [pMTL83251] (circles), *C. saccharoperbutylacetonicum* [pMTL83251_$P_{pta\text{-}ack}$_FAST] (triangles), and *C. saccharoperbutylacetonicum* [pMTL83251_P_{bgaL}_FAST] without (open squares) and with (filled squares) induction of gene expression using 20 mM lactose. **b** growth (solid lines) and fluorescence intensities (dotted lines) of *C. saccharoperbutylacetonicum* [pMTL83251_P_{thlA}_FAST] (diamonds), *C. saccharoperbutylacetonicum* [pMTL83251_P_{bld}_FAST] (downward-facing triangles), and *C. saccharoperbutylacetonicum* [pMTL83251_P_{lctB}_FAST] without (open left-facing triangles) or with induction of gene expression using 15 mM *L*-lactate (half-filled left-facing triangles) or 15 mM *D*-lactate (filled left-facing triangles). Black dashed line indicates time of induction of P_{bgaL} or P_{lctB}. Error bars indicate standard deviations, n=3

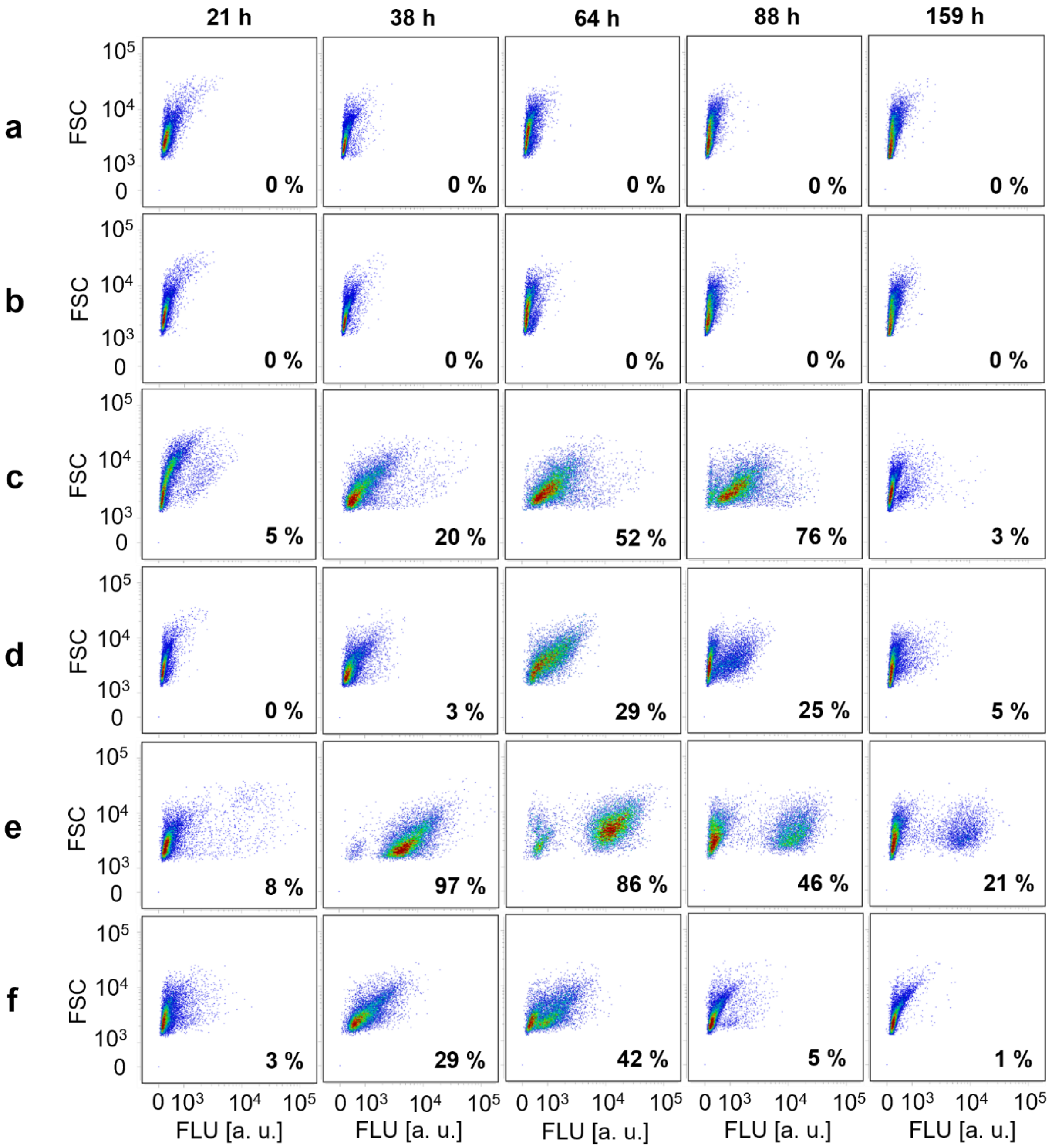

Fig. S4 Results of flow cytometric measurements with FAST-producing *C. saccharoperbutylacetonicum* strains. Displayed are density plots of *C. saccharoperbutylacetonicum* [pMTL83251] (a), *C. saccharoperbutylacetonicum* [pMTL83251_P_{tcdB}_FAST] (b), induced *C. saccharoperbutylacetonicum* [pMTL83251_P_{bgaL}_FAST] (c), induced *C. saccharoperbutylacetonicum* [pMTL83251_P_{tcdB}_FAST_P_{bgaL}_tcdR] (d), *C. saccharoperbutylacetonicum* [pMTL83251_P_{bld}_FAST] (e), and *C. saccharoperbutylacetonicum* [pMTL83251_P_{tcdB}_FAST_P_{bld}_tcdR] (f) after 21 h, 38 h, 64 h, 88 h, and 159 h of cultivation, respectively. Cells were supplemented with 5 µM ^{TF}Lime. Amounts of fluorescent cells are given in % in each density plot. Induction of strains harboring P_{bgaL} was achieved by addition of 20 mM lactose

ATGTCAGAAGAATCATTAGTTTTATCAACAATTGAAGGACCAATTGCAATTTTAACATTAAATAGACC
ACAAGCATTAAATGCATTATCACCAGCATTAATTGATGATTTAATTAGACATTTAGAAGCATGTGATG
CAGATGATACAATTAGAGTTATTATTATTACAGGAGCAGGAAGAGCATTTGCAGCAGGAGCAGATAT
TAAAGCAATGGCAAATGCAACACCAATTGATATGTTAACATCAGGAATGATTGCAAGATGGGCAAG
AATTGCAGCAGTTAGAAAACCAGTTATTGCAGCAGTTAATGGATATGCATTAGGAGGAGGATGTGA
ATTAGCAATGATGTGTGATATTATTATTGCATCAGAAAATGCACAATTTGGACAACCAGAAATTAATT
TAGGAATTATTCCAGGAGCAGGAGGAACACAAAGATTAACAAGAGCATTAGGACCATATAGAGCAA
TGGAATTAATTTTAACAGGAGCAACAATTTCAGCACAAGAAGCATTAGCACATGGATTAGTTTGTAG
AGTTTGTCCACCAGAATCATTATTAGATGAAGCAAGAAGAATTGCACAAACAATTGCAACAAAATCA
CCATTAGCAGTTCAATTAGCAAAAGAAGCAGTTAGAATGGCAGCAGAAACAACAGTTAGAGAAGGA
TTAGCAATTGAATTAAGAAATTTTTATTTATTATTTGCATCAGCAGATCAAAAAGAAGGAATGCAAGC
ATTTATTGAAAAAAGAGCACCAAATTTTTCAGGAAGATAA

Fig. S5 Nucleotide sequence of the *ehy* gene from *Chloroflexus aurantiacus* codon-optimized for *A. woodii* (*ehy_opt*).
